# Epigenetic alterations in TRAMP mice: epigenome DNA methylation profiling using MeDIP-seq

**DOI:** 10.1186/s13578-018-0201-y

**Published:** 2018-01-12

**Authors:** Wenji Li, Ying Huang, Davit Sargsyan, Tin Oo Khor, Yue Guo, Limin Shu, Anne Yuqing Yang, Chengyue Zhang, Ximena Paredes-Gonzalez, Michael Verzi, Ronald P. Hart, Ah-Ng Kong

**Affiliations:** 1 Center for Phytochemical Epigenome Studies, Ernest Mario School of Pharmacy, The State University of New Jersey, Piscataway, NJ 08854 USA; 2 Department of Pharmaceutics, Ernest Mario School of Pharmacy, Rutgers, The State University of New Jersey, 160 Frelinghuysen Road, Piscataway, NJ 08854 USA; 3 Graduate Program in Pharmaceutical Sciences, Ernest Mario School of Pharmacy, The State University of New Jersey, Piscataway, NJ 08854 USA; 4 Department of Genetics, The State University of New Jersey, Piscataway, NJ 08854 USA; 5 Department of Cell Biology and Neuroscience, Rutgers, The State University of New Jersey, Piscataway, NJ 08854 USA

**Keywords:** MeDIP-seq, Epigenetics, DNA methylation, TRAMP, Prostate cancer

## Abstract

**Purpose:**

We investigated the genomic DNA methylation profile of prostate cancer in transgenic adenocarcinoma of the mouse prostate (TRAMP) cancer model and to analyze the crosstalk among targeted genes and the related functional pathways.

**Methods:**

Prostate DNA samples from 24-week-old TRAMP and C57BL/6 male mice were isolated. The DNA methylation profiles were analyzed by methylated DNA immunoprecipitation (MeDIP) followed by next-generation sequencing (MeDIP-seq). Canonical pathways, diseases and function and network analyses of the different samples were then performed using the Ingenuity^®^ Pathway Analysis (IPA) software. Some target genes with significant difference in methylation were selected for validation using methylation specific primers (MSP) and qPCR.

**Results:**

TRAMP mice undergo extensive aberrant CpG hyper- and hypo-methylation. There were 2147 genes with a significant (log2-change ≥ 2) change in CpG methylation between the two groups, as mapped by the IPA software. Among these genes, the methylation of 1105 and 1042 genes was significantly decreased and increased, respectively, in TRAMP prostate tumors. The top associated disease identified by IPA was adenocarcinoma; however, the cAMP response element-binding protein (CREB)-, histone deacetylase 2 (HDAC2)-, glutathione S-transferase pi (GSTP1)- and polyubiquitin-C (UBC)-related pathways showed significantly altered methylation profiles based on the canonical pathway and network analyses. MSP and qPCR results of genes of interests corroborated with MeDIP-seq findings.

**Conclusions:**

This is the first MeDIP-seq with IPA analysis of the TRAMP model to provide novel insight into the genome-wide methylation profile of prostate cancer. Studies on epigenetics, such as DNA methylation, will potentially provide novel avenues and strategies for further development of biomarkers targeted for treatment and prevention approaches for prostate cancer.

## Background

Prostate cancer is the second leading male cancer (accounts for 13.8% of all male cancers) and its prevalence ranking number five among all cancers [1]. In the United States, prostate cancer is the most common male cancer subtype, apart from non-melanoma skin cancer [2]. Prostate cancer is a clinically heterogeneous disease with marked variability in patient outcomes [3]. Early detection, accurate prediction and successful management of prostate cancer represent some of the most challenging and controversial issues [4]. Interestingly, epigenetic changes are hallmarks of prostate cancer, among which DNA methylation is the most frequently studied [5].

Epigenetic changes include DNA methylation, histone modification, and posttranslational gene regulation by micro-RNAs (miRNAs) [6]. Among these, DNA methylation has been well studied, and aberrant DNA methylation patterns are a characteristic feature of cancer [7–9]. The first reported epigenetic changes in human cancer were DNA methylation losses. Since then, genomic hypomethylation has been found to be associated with multiple cancer types [10, 11]. In addition, hypermethylation of CpG islands (CGIs) at promoters of tumor suppressor genes, homeobox genes and other sequences are other consistent epigenetic features of cancer [12, 13]. CpG island methylator-phenotype (CIMP) tumors have been identified in many cancers, including oral cancer, colorectal cancer [14] and colon cancer [15]. Therefore, it is worthwhile to profile the global DNA methylation changes between cancer models and controls to elucidate the mechanisms of carcinogenesis.

The transgenic adenocarcinoma of the mouse prostate (TRAMP) model closely represents the pathogenesis of human prostate cancer because male TRAMP mice spontaneously develop autochthonous prostate tumors following the onset of puberty [16] and it specifically induces transgene expression in the prostate, displays distant organ metastases and it has castration-resistant properties [17]. DNA methylation in the TRAMP model has been widely studied in vitro and in vivo, resulting in the discovery of the methylated markers Nuclear factor (erythroid-derived 2)-like 2(NRF2) [18], O6-alkylguanine DNA alkyltransferase (MGMT) [19], glutathione S-transferase pi (GSTP1) [20], 14-3-3σ [21], and Krueppel-like factor 6 (KLF6) [22].

However, only Shannon et al. have compared global methylation alteration among TRAMP and wild type (WT) mice [23]. Systemic comparisons and analyses of the genomic methylation status of prostate cancer models and normal controls are needed to determine the underlying interactions between these target genes and to discover new biomarkers. We are the first to perform methylated DNA immunoprecipitation (MeDIP) coupled with next-generation sequencing (MeDIP-seq) followed by Ingenuity^®^ Pathway Analysis (IPA) studies to investigate the crosstalk among important genes and to analyze overlapping functional pathways by comparing the whole genomic DNA methylation patterns between the TRAMP model and controls.

## Methods

### Genomic DNA extraction from TRAMP and C57BL/6 male mice

The breeding of TRAMP mice was same as for our previous studies [24, 25]. Briefly, female hemizygous C57BL/TGN TRAMP mice, line PB Tag 8247NG (Jackson Laboratory, Bar Harbor, ME), were bred with the same genetic background male C57BL/6 mice (Jackson Laboratory, Bar Harbor, ME). Identity of transgenic mice was established by PCR-based DNA genotyping using the primers suggested by The Jackson Laboratory as we previously described [24, 25]. F1 (first generation from cross breeding) or F2 (second generation from cross breeding) male TRAMP mice were used for the studies. Mice were housed in cages containing wood-chip bedding in a temperature-controlled room (20–22 °C) with a 12-h-light/dark cycle and a relative humidity of 45–55% at Rutgers Animal Care Facility. All animals received water and food ad libitum until sacrifice (24 weeks of age) by carbon dioxide euthanasia. The study was performed using an IACUC-approved protocol (01-016) at Rutgers University. Mice were weighed and evaluated in the overall health twice weekly throughout the study. Presences of palpable tumor, metastases, genitourinary (GU) apparatus weight were evaluated upon necropsy and prostate intraepithelial neoplasia lesions (evaluated by H&E staining) were monitored in the TRAMP group (data not shown). Prostate samples from three 24-week-old TRAMP and three 24 weeks old C57BL/6 mice (maintained under similar conditions) were randomly selected out. A DNeasy Kit (Qiagen, Valencia, CA, USA) was used to extract the genomic DNA (gDNA) from whole prostate samples of three 24-week-old male TRAMP mice and three age-matched C57BL/6 male mice following the kit’s protocol. After extraction and purification, the gDNA samples were electrophoresed on an agarose gel, and the OD ratios were measured to confirm the purity and concentrations of the gDNA prior to fragmentation by Covaris (Covaris, Inc., Woburn, MA USA). The fragmented gDNA was then evaluated for size distribution and concentration using an Agilent Bioanalyzer 2100 and a NanoDrop spectrophotometer.

### MeDIP-seq measurement

Following the manufacturer’s instructions, MeDIP was performed to analyze genome-wide methylation using the MagMeDIP Kit from Diagenode (Diagenode Inc., Denville, NJ, USA). Methylated DNA was separated from unmethylated fragments by immunoprecipitation with a 5-methylcytidine monoclonal antibody from Eurogentec (Eurogentec S.A., Seraing, Belgium). Illumina libraries were then created from the captured gDNA using NEBNext reagents (New England Biolabs, Ipswich, MA, USA). Enriched libraries were evaluated for size distribution and concentration using an Agilent Bioanalyzer 2100, and the samples were then sequenced on an Illumina HiSeq2000 machine, which generated paired-end reads of 90 or 100 nucleotides (nt). The results were analyzed for data quality and exome coverage using the platform provided by DNAnexus (DNAnexus, Inc., Mountain View, CA, USA). The samples were sent to Otogenetics Corp. (Norcross, GA) for Illumina sequencing and alignment with the reference mouse genome. The resulting BAM files were downloaded for analysis.

Modified from the Trapnell method, the MeDIP alignments were compared with control sample alignments using Cuffdiff 2.0.2 with no length correction [26]. A list of overlapping regions of sequence alignment that were common to both the immunoprecipitated and control samples was created and used to determine the quantitative enrichment of the MeDIP samples over the control samples using Cuffdiff; statistically significant peaks (reads) at a 5% false discovery rate (FDR) and a minimum fourfold difference, as calculated using the Cummerbund package in R, were selected [26]. Sequencing reads were matched with the adjacent annotated genes using ChIPpeakAnno [27], and the uniquely mapped reads were used to compare the differences between TRAMP and wild-type mice.

The reads were visualized and individual genes examined using Integrative Genomics Viewer (IGV) [28]. IGV allows users to explore aligned reads at any level of details by changing resolution, scrolling through and searching for specific chromosomes, genes or regions [29]. We specifically examined genes that produced differences in methylation between the TRAMP and control groups of fourfold or more (log2 difference ≥ 2). IGV provided more in-depth understanding of these differences by graphing distributions of reads against the reference genome. Heat maps were used to graphically represent methylation levels in genes and to compare the methylation of the two groups. We used green color to signify positive differences in methylation and the red color for the negative differences (TRAMP minus control). Brighter shades correspond to more extreme values, i.e. larger fold-changes.

### Canonical pathways, diseases and function and network analysis by IPA

Genes selected from the MeDIP-seq experiment based on significantly increased or decreased fold changes (log2-fold change ≥ 2) in methylation were analyzed (based on the p values; TRAMP vs control) using IPA 4.0 When using IPA (IPA 4.0, ingenuity systems, http://www.ingenuity.com), the pathway enrichment *p* value is calculated using the right-tailed Fisher’s exact test. A smaller *p* value indicated that the association was less likely to be random and more likely to be significant. In general, values of 0.05 (for *p* value) or 1.30 (for − log_10_p) were set as the thresholds. p values less than 0.05 or − log_10_p more than 1.30 were considered to be statistically significant, non-random associations. IPA utilized gene symbols to identify neighboring enriched methylation peaks using ChIPpeakAnno for all of the analyses. Using IPA, 2147 genes from TRAMP group that showed a log2-fold change ≥ 2 compared with the control group were mapped. Based on these fold changes, IPA identified the canonical pathways, biological functions/related diseases and networks that were closely related to the TRAMP model.

### MeDIP-seq data validation via methylation-specific PCR (MSP)

Genomic DNA was extracted and purified from six prostate samples (three from TRAMP mice and three from normal C57BL/6 mice) using the AllPrep DNA/RNA/Protein Mini Kit (Qiagen, Valencia, CA, USA). Then 500 ng genomic DNA was underwent bisulfite conversion with an EZ DNA Methylation-Gold Kit (Zymo Research Corp., Orange, CA) following the kit’s protocol as described previously [30]. The converted DNA was amplified by PCR using EpiTaq HS DNA polymerase (Clontech Laboratories Inc, Mountain View, CA 94043, USA). According to MeDIP-seq results, four target genes (two with increased methylation and two with decreased methylation), dynein cytoplasmic 1 intermediate chain 1 (DYNC1I1), solute carrier family 1 member 4 (SLC1A4), XRCC6-binding protein 1 (Xrcc6bp1) and transthyretin (TTR), were selected for MSP validation. The primers’ sequences for the methylated reactions (MF and MR) and for the unmethylated reactions (UF and UR) and band size of products are listed in Table 1. By running agarose gel electrophoresis, the amplification product bands were isolated and were semi-quantitated by densitometry using ImageJ (Version 1.48d; NIH, Bethesda, Maryland, USA).

**Table 1:** Primer sequences used in MSP

Gene name	Primer name	Primer sequence	Band size (bp)
Dync1i1	Dync1i1-MF	TATGAAGAAAAATATAGTAAGATACGG	232
	Dync1i1-MR	ACGAACATTTCACATTTCGAA	
	Dync1i1-UF	TTTATGAAGAAAAATATAGTAAGATATGG	235
	Dync1i1-UR	CACAAACATTTCACATTTCAAA	
Slc1a4	Slc1a4-MF	ATAAATTATTTTTTTTATGTTACGG	216
	Slc1a4-MR	TTAATAATACATACCTATAATCCGAC	
	Slc1a4-UF	ATAAATTATTTTTTTTATGTTATGG	216
	Slc1a4-UR	TTAATAATACATACCTATAATCCAAC	
Xrcc6bp1	Xrcc6bp1-MF	GTTAATGTGAGAGTTAGAATAGTATAGGAC	110
	Xrcc6bp1-MR	AATTAATACAATATTTCGATACCGAT	
	Xrcc6bp1-UF	GTTAATGTGAGAGTTAGAATAGTATAGGAT	110
	Xrcc6bp1-UR	AATTAATACAATATTTCAATACCAAT	
TTR	TTR-MF	GGAATTTAAGATACGGTTTATATCGA	106
	TTR-MR	AACACTCTTTCGAACATACTCGAC	
	TTR-UF	AGGAATTTAAGATATGGTTTATATTGA	108
	TTR-UR	AAACACTCTTTCAAACATACTCAAC	

Primer sequences are started from 5′ (left) to 3′ (right)

*MF* forward primer sequence for the methylated reactions, *MR* reverse primer sequence for the methylated reactions, *UF* forward primer sequence for the unmethylated reactions, *UR* reverse primer sequence for the unmethylated reactions

### Validation of selected gene expression by quantitative real-time RT-PCR

Total RNA was extracted and purified from six prostate samples (three from TRAMP mice and three from normal C57BL/6 mice) using the same kit above. cDNA was synthesized from total RNA using a SuperScript III First-Strand Synthesis System (Invitrogen, Grand Island, NY) following the kit’s instruction. mRNA levels were determined using quantitative real-time PCR (qPCR). Histamine *N*-methyltransferase (HNMT), Dync1i1, SLC1A4, crystallin zeta (CRYZ) and TTR were randomly selected to compare mRNA expression among WT and TRAMP mice prostate samples. The primers’ sequences for HNMT, DYNC1I1, SLC1A4, CRYZ, TTR and β-Actin are listed in Table 2.

**Table 2:** Primer sequences used in qPCR

Gene name	Primer name	Primer sequence
HNMT	Sense	5′-GCTGCCAGTGCTAAAATTCTC-3′
	Antisense	5′-CAGGTCATCCAGTATCTGCG-3′
DYNC1I1	Sense	5′-GTGTACGATGTCATGTGGTCC-3′
	Antisense	5′-AACTCGGTTTAG GGCAGATG-3′
SLC1A4	Sense	5′-CCTCACAATTGCCATCATCTT G-3′
	Antisense	5′-CATCCCCTTCCACATTCACC-3′
CRYZ	Sense	5′-GCAGCCGATGACACTATCTAC-3′
	Antisense	5′-GCCCCATGAACCAAAACG-3′
TTR	Sense	5′-AATCGTACTGGAAGACACTTGG-3′
	Antisense	5′-TGGTGCTGTAGGAGTATGG-3′
β-Actin	Sense	5′-CGTTCAATACCCCAGCCATG-3′
	Antisense	5′-ACCCCGTCACCAGAGTCC-3′

## Results

### MeDIP-seq results comparison

**Fig. 1:**
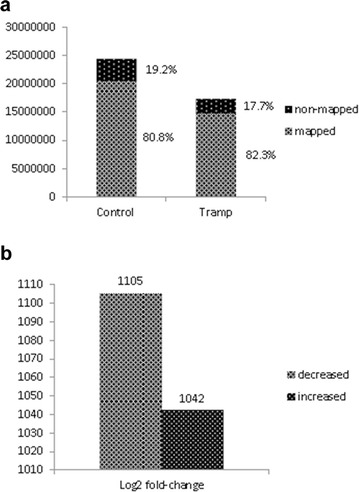
Total mapping reads in the control and TRAMP mice (**a**) and the total number of significantly (log2-fold change ≥ 2) increased and decreased methylated genes in the TRAMP mice compared with the control mice (**b**)

One of our main goals of this study was to screen and reveal aberrantly methylated genes to discover the related functions and pathways that might mediate the development of prostate cancer. To accomplish this goal in an unbiased manner, the MeDIP-seq results were analyzed using IPA. The first objective was to compare the total number of molecules with altered methylation in prostate samples of TRAMP mice to that of normal mice. Prostate samples were collected from the TRAMP and C57BL/6 mice, gDNA was isolated, and whole-genome DNA methylation analysis was performed using the described MeDIP-seq method. The results were analyzed in a paired manner, comparing the prostate tissue samples for each model. For the control, 16 509 344 (80.8%) mapped and 3 921 684 (19.2%) unmapped reads, for a total of 20 431 028 reads, were obtained. For the TRAMP mice, 12 097 771 (82.3%) mapped and 2 609 269 (17.7%) unmapped reads, for a total of 14 707 040 reads, were obtained (Fig. 1a). After identification and mapping to the library, the identified methylated regions (peaks) of the given genes were compared between the TRAMP and control mice, and IPA was used to identify the genes with significantly altered methylation in the TRAMP mice compared with the controls (*p* < 0.05 or − log_10_p > 1.30, and log2-fold change ≥ 2).

**Fig. 2:**
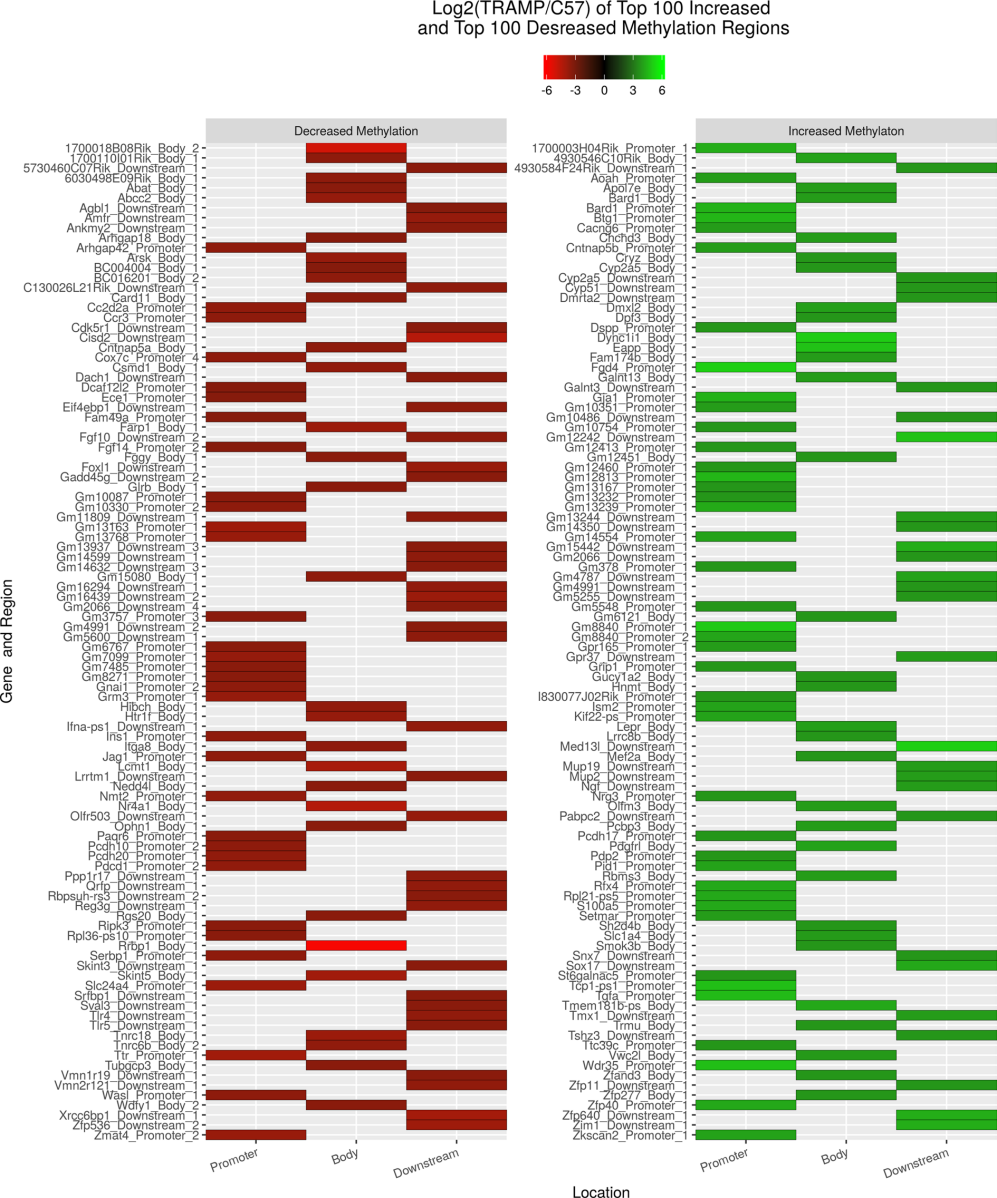
Heat-map of top 100 decreased or increased (log2-fold change) methylated genes comparing with TRAMP to WT in different regions by MeDIP analysis, ranked by alphabetic

Genes were sorted in the order of differences in methylation. Genes with the change in methylation levels of fourfold or more (both, positive and negative) were then used as an input to the IPA software. According to the IPA setting, the *p* value for a given process annotation was calculated by considering (1) the number of focus genes that participated in the process and (2) the total number of genes that are known to be associated with that process in the selected reference set. The more focus genes that are involved, the more likely the association is not due to random chance, resulting in a more significant p value (larger − log_10_p value). Altogether, 2147 genes between the two groups showed a significant change (log2-fold change ≥ 2) in methylated peaks. Compared with the control, significantly decreased methylation of 1105 genes and significantly increased methylation of 1042 genes were observed in TRAMP (Fig. 1b). The top fifty genes with increased methylation (Table 3) or decreased methylation (Table 4) located in promoter region, gene body or downstream of the gene were highlighted according to the log2-fold change, ranking from the largest to the smallest change and with significant statistic difference (*p* < 0.05). We also plotted the top 100 decreased or increased (log2-fold change) methylated genes comparing with TRAMP to WT in different regions by MeDIP analysis, ranked by alphabet (Fig. 2).

**Table 3:** Top 50 annotated genes with increased methylation, ranked by log2-fold change

Rank	Symbol	Gene name	Log2-fold change (TRAMP/WT)	Location	Type(s)	Methylation region
1	FGD4	FYVE, RhoGEF and PH domain containing 4	4.993	Cytoplasm	Other	Promoter
2	MED13L	Mediator complex subunit 13-like	4.993	Nucleus	Other	Downstream
3	DYNC1I1	Dynein, cytoplasmic 1, intermediate chain 1	4.926	Cytoplasm	Other	Body
4	XK	X-linked Kx blood group	4.781	Plasma membrane	Transporter	Body
5	EAPP	E2F-associated phosphoprotein	4.703	Cytoplasm	Other	Body
6	TGFA	Transforming growth factor, alpha	4.534	Extracellular space	Growth factor	Promoter
7	BTG1	B-cell translocation gene 1, anti-proliferative	4.440	Nucleus	Transcription regulator	Promoter
8	BARD1	BRCA1 associated RING domain 1	4.341	Nucleus	Transcription regulator	Promoter
9	GJA1	Gap junction protein, alpha 1, 43 kDa	4.341	Plasma membrane	Transporter	Promoter
10	Zfp640	Zinc finger protein 640	4.234	Other	Other	Downstream
11	S100A5	S100 calcium-binding protein A5	4.119	Nucleus	Other	Promoter
12	SOX17	SRY (sex-determining region Y)-box 17	4.119	Nucleus	Transcription regulator	Downstream
13	PDGFRL	Platelet-derived growth factor receptor-like	3.993	Plasma membrane	Kinase	Body
14	ZKSCAN2	Zinc finger with KRAB and SCAN domains 2	3.993	Nucleus	Transcription regulator	Promoter
15	DMXL2	Dmx-like 2	3.926	Cytoplasm	Other	Body
16	LEPR	Leptin receptor	3.926	Plasma membrane	Transmembrane receptor	Body
17	AOAH	Acyloxyacyl hydrolase (neutrophil)	3.855	Extracellular space	Enzyme	Promoter
18	Apol7e	Apolipoprotein L 7e	3.855	Other	Other	Body
19	CACNG6	Calcium channel, voltage-dependent, gamma subunit 6	3.855	Plasma membrane	Ion channel	Promoter
20	CHCHD3	Coiled-coil-helix-coiled-coil-helix domain containing 3	3.855	Cytoplasm	Other	Body
21	FAM174B	Family with sequence similarity 174, member B	3.855	Other	Other	Body
22	GALNT13	Polypeptide *N*-acetylgalactosaminyltransferase 13	3.855	Cytoplasm	Enzyme	Body
23	GPR37	G protein-coupled receptor 37 (endothelin receptor type B-like)	3.855	Plasma membrane	G-protein coupled receptor	Downstream
24	Mup1	Major urinary protein 1	3.855	Extracellular space	Other	Downstream
25	NGF	Nerve growth factor (beta polypeptide)	3.855	Extracellular space	Growth factor	Downstream
26	OLFM3	Olfactomedin 3	3.855	Cytoplasm	Other	Body
27	PCBP3	Poly(rC)-binding protein 3	3.855	Nucleus	Other	Body
28	RBMS3	RNA-binding motif, single-stranded-interacting protein 3	3.855	Other	Other	Body
29	TMX1	Thioredoxin-related transmembrane protein 1	3.855	Cytoplasm	Enzyme	Downstream
30	ZNF14	Zinc finger protein 14	3.855	Nucleus	Transcription regulator	Body
31	SLC1A4	Solute carrier family 1 (glutamate/neutral amino acid transporter), member 4	3.807	Plasma membrane	Transporter	Body
32	ZFAND3	Zinc finger, AN1-type domain 3	3.717	Other	Other	Body
33	C1orf162	Chromosome 1 open reading frame 162	3.703	Other	Transporter	Promoter
34	C9orf131	Chromosome 9 open reading frame 131	3.703	Other	Other	Body
35	CRYZ	Crystallin, zeta (quinone reductase)	3.703	Cytoplasm	Enzyme	Body
36	CYP2A6	Cytochrome P450, family 2, subfamily A, polypeptide 6	3.703	Cytoplasm	Enzyme	Body
37	CYP51A1	Cytochrome P450, family 51, subfamily A, polypeptide 1	3.703	Cytoplasm	Enzyme	Downstream
38	DSPP	Dentin sialophosphoprotein	3.703	Extracellular space	Other	Promoter
39	GALNT3	Polypeptide *N*-acetylgalactosaminyltransferase 3	3.703	Cytoplasm	Enzyme	Downstream
40	Gm4836	Predicted gene 4836	3.703	Nucleus	Other	Downstream
41	GRIP1	Glutamate receptor-interacting protein 1	3.703	Plasma membrane	Transcription regulator	Promoter
42	GUCY1A2	Guanylate cyclase 1, soluble, alpha 2	3.703	Cytoplasm	Enzyme	Body
43	HNMT	Histamine *N*-methyltransferase	3.703	Cytoplasm	Enzyme	Body
44	LRRC8B	Leucine-rich repeat containing 8 family, member B	3.703	Other	Other	Body
45	MEF2A	Myocyte enhancer factor 2A	3.703	Nucleus	Transcription regulator	Body
46	NRG3	Neuregulin 3	3.703	Extracellular space	Growth factor	Promoter
47	PCDH17	Protocadherin 17	3.703	Other	Other	Promoter
48	PDP2	Pyruvate dehydrogenase phosphatase catalytic subunit 2	3.703	Cytoplasm	Phosphatase	Promoter
49	SH2D4B	SH2 domain containing 4B	3.703	Other	Other	Body
50	Smok2b	Sperm motility kinase 2B	3.703	Other	Kinase	Body

**Table 4:** Top 50 annotated genes with decreased methylation, ranked by log2-fold change

Rank	Symbol	Gene name	Log2 fold change (TRAMP/WT)	Location	Type(s)	Methylation region
1	Rrbp1	Ribosome-binding protein 1	− 5.824	Cytoplasm	Transporter	Body
2	CISD2	CDGSH iron sulfur domain 2	− 4.373	Cytoplasm	Other	Downstream
3	NR4A1	Nuclear receptor subfamily 4, group A, member 1	− 4.324	Nucleus	Ligand-dependent nuclear receptor	Body
4	LCMT1	Leucine carboxyl methyltransferase 1	− 4.051	Cytoplasm	Enzyme	Body
5	XRCC6BP1	XRCC6 binding protein 1	− 3.990	Other	Kinase	Downstream
6	TTR	Transthyretin	− 3.926	Extracellular space	Transporter	Promoter
7	ZNF536	Zinc finger protein 536	− 3.859	Other	Other	Downstream
8	FARP1	FERM, RhoGEF (ARHGEF) and pleckstrin domain protein 1 (chondrocyte-derived)	− 3.788	Plasma membrane	Other	Body
9	TNRC18	Trinucleotide repeat containing 18	− 3.788	Other	Other	Body
10	FOXL1	Forkhead box L1	− 3.714	Nucleus	Transcription regulator	Downstream
11	ZMAT4	Zinc finger, matrin-type 4	− 3.714	Nucleus	Other	Promoter
12	ABCC2	ATP-binding cassette, sub-family C (CFTR/MRP), member 2	− 3.636	Plasma membrane	Transporter	Body
13	AMFR	Autocrine motility factor receptor, E3 ubiquitin protein ligase	− 3.636	Plasma membrane	Transmembrane receptor	Downstream
14	ARSK	Arylsulfatase family, member K	− 3.636	Extracellular space	enzyme	Body
15	GRM3	Glutamate receptor, metabotropic 3	− 3.636	Plasma membrane	G-protein coupled receptor	Promoter
16	HTR1F	5-hydroxytryptamine (serotonin) receptor 1F, G protein-coupled	− 3.636	Plasma membrane	G-protein coupled receptor	Body
17	CC2D2A	Coiled-coil and C2 domain containing 2A	− 3.554	Other	Other	Promoter
18	CSMD1	CUB and Sushi multiple domains 1	− 3.554	Plasma membrane	Other	Body
19	HIBCH	3-Hydroxyisobutyryl-CoA hydrolase	− 3.554	Cytoplasm	Enzyme	Body
20	NMT2	*N*-Myristoyltransferase 2	− 3.554	Cytoplasm	Enzyme	Promoter
21	PCDH20	Protocadherin 20	− 3.554	Other	Other	Promoter
22	PDCD1	Programmed cell death 1	− 3.554	Plasma membrane	Phosphatase	Promoter
23	QRFP	Pyroglutamylated RFamide peptide	− 3.554	Extracellular space	Other	Downstream
24	REG3G	Regenerating islet-derived 3 gamma	− 3.554	Extracellular space	Other	Downstream
25	TLR4	Toll-like receptor 4	− 3.554	Plasma membrane	Transmembrane receptor	Downstream
26	TNRC6B	Trinucleotide repeat containing 6B	− 3.554	Other	Other	Body
27	CCR3	Chemokine (C–C motif) receptor 3	− 3.466	Plasma membrane	G-protein coupled receptor	Promoter
28	Cngb1	Cyclic nucleotide gated channel beta 1	− 3.466	Other	Other	Body
29	CNTNAP5	Contactin associated protein-like 5	− 3.466	Other	Other	Body
30	Cox7c	Cytochrome *c* oxidase subunit VIIc	− 3.466	Cytoplasm	Other	Promoter
31	EIF4EBP1	Eukaryotic translation initiation Factor 4E binding protein 1	− 3.466	Cytoplasm	Translation regulator	Downstream
32	FGF10	Fibroblast growth factor 10	− 3.466	Extracellular space	Growth factor	Downstream
33	GNAI1	Guanine nucleotide-binding protein (G protein), alpha inhibiting activity polypeptide 1	− 3.466	Plasma membrane	Enzyme	Promoter
34	Ins1	Insulin I	− 3.466	Extracellular space	Other	Promoter
35	ITGA8	Integrin, alpha 8	− 3.466	Plasma membrane	Other	Body
36	JAG1	Jagged 1	− 3.466	Extracellular space	Growth factor	Promoter
37	Pcdh10	Protocadherin 10	− 3.466	Other	Other	Promoter
38	PPP1R17	Protein phosphatase 1, regulatory subunit 17	− 3.466	Cytoplasm	Other	Downstream
39	Serbp1	Serpine1 mRNA-binding protein 1	− 3.466	Cytoplasm	Other	Promoter
40	Wasl	Wiskott-Aldrich syndrome-like (human)	− 3.466	Cytoplasm	Other	Promoter
41	ABAT	4-Aminobutyrate aminotransferase	− 3.373	Cytoplasm	Enzyme	Body
42	ANKMY2	Ankyrin repeat and MYND domain containing 2	− 3.373	Plasma membrane	Other	Downstream
43	Card11	Caspase recruitment domain family, member 11	− 3.373	Other	Other	Body
44	CDK5R1	Cyclin-dependent kinase 5, regulatory subunit 1 (p35)	− 3.373	Nucleus	Kinase	Downstream
45	DACH1	Dachshund family transcription factor 1	− 3.373	Nucleus	Transcription regulator	Downstream
46	FGGY	FGGY carbohydrate kinase domain containing	− 3.373	Other	Other	Body
47	GADD45G	Growth arrest and DNA-damage-inducible, gamma	− 3.373	Nucleus	Other	Downstream
48	GLRB	Glycine receptor, beta	− 3.373	Plasma membrane	Ion channel	Body
49	LRRTM1	Leucine-rich repeat transmembrane neuronal 1	− 3.373	Plasma membrane	Other	Downstream
50	NEDD4L	Neural precursor cell expressed, developmentally down-regulated 4-like, E3 ubiquitin protein ligase	− 3.373	Cytoplasm	Enzyme	Body

**Fig. 3:**
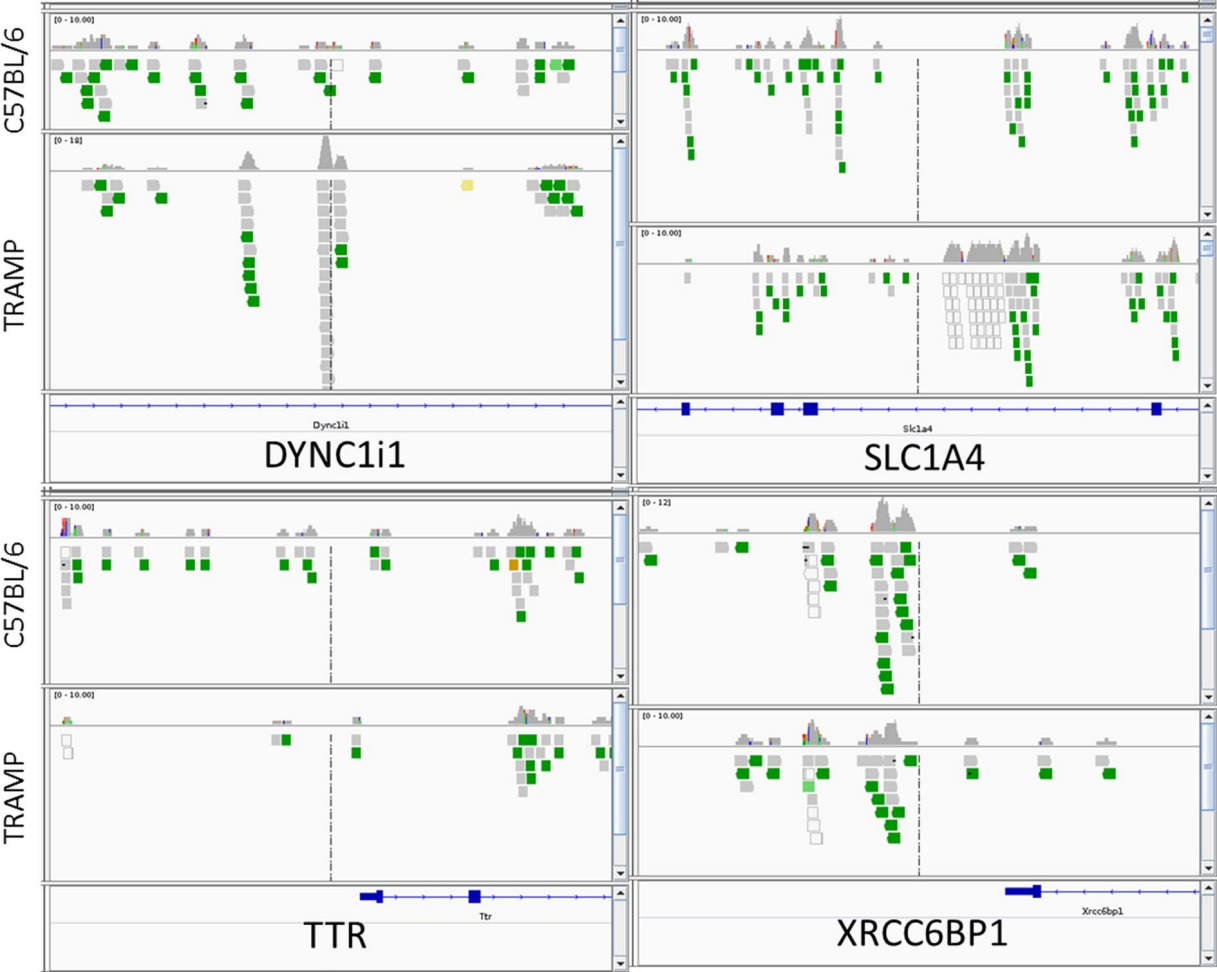
integrative genomics viewer visualization of the aligned reads’ distribution against reference genome for four targeted genes: DYNC1i1, SLC1A4, TTR and XRCC6BP1

Four genes of interest, DYNC1I1, SLC1A4, XRCC6BP1 and TTR were analyzed by IGV (Fig. 3), which provides more in-depth understanding of these differences between TRAMP and control mice. The IGV results are in accordance with the MeDIP-seq finding. In TRAMP mice, the methylation ratio of DYNC1I1 and SLC1A4 were increased, whereas the methylation ratio of TTR and XRCC6BP1 were decreased. The methylation results have been validated by MSP.

These results demonstrate a fundamental difference in the global pattern of gene methylation between the TRAMP prostate tumor and control prostate tissue. The potential impact of this difference was further assessed using IPA by analyzing the canonical pathways, diseases and functions, and networks related to these methylation changes.

### MeDIP-seq data validation by MSP

According to the MeDIP-seq results, four interesting genes, two with increased methylation (TRAMP vs WT), DYNC1I1 and SLC1A4, and two with decreased methylation (TRAMP vs WT), XRCC6BP1 and TTR were selected to carry out MSP to validate the MeDIP-seq data. MSP results indicated a similar trend in agreement with the MeDIP-seq results.

**Fig. 4:**
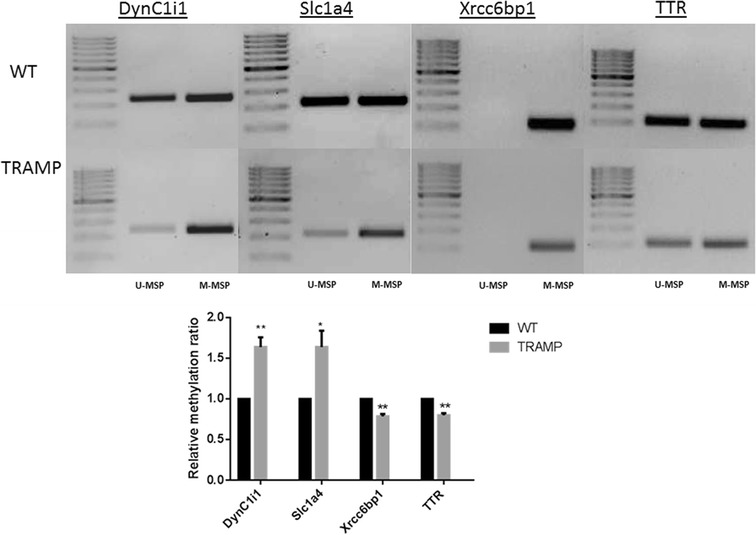
Medip-Seq Validation by methylation-specific PCR (MSP). Representative electrophoretogram is presented in the top panel. M-MSP: methylated reaction of MSP, U-MSP: unmethylated reaction of MSP. The relative intensity of the methylated and unmethylated band was measured by ImageJ and presented in the bottom panel. All of the data are presented as the mean ± SD. *p < 0.05 vs the control WT group

The results showed, in Dync1i1 and Slc1a4 genes, the relative density of M-MSP (methylated MSP) to that of U-MSP (unmethylated MSP) in TRAMP group were increased, which indicated that the CpG sites of these genes were hypermethylated in TRAMP mice (Fig. 4). Similarly, in Xrcc6bp1 and TTR, the relative density of M-MSP to that of U-MSP in TRAMP group was decreased, which indicated that the CpG sites of these genes were hypomethylated in TRAMP mice (Fig. 4).

### qPCR validation of selected gene expression

**Fig. 5:**
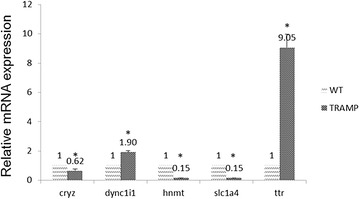
Comparison of mRNA expression of CRYZ, DYNC1I1, HNMT, SLC1A4 and TTR among WT and TRAMP mice prostate samples. Total mRNA was isolated and analyzed using quantitative real-time PCR. The data are presented as the mean ± SD of three independent experiments. *p < 0.05 vs the control WT group

When mRNA levels were measured by qPCR, the relative expression levels of CRYZ, DYNC1I1, HNMT, SLC1A4 and TTR in TRAMP group were 0.62, 1.90, 0.15, 0.15 and 9.05 fold compared with WT (Fig. 5). Among these, TTR expression was increased by 9.05-fold over WT, which agreed with results reported by Wang et al. [31] that expression levels of TTR were significantly higher in prostate cancer tissue than in normal and benign prostate hyperplasia tissue. When comparing mRNA expression and Methylation validation results, reciprocal relationships were found in TTR in TRAMP, which indicated decreased methylation in promoter region but increased gene expression when comparing with WT. In contrast, DNA methylation in the gene body or downstream may or may not follow a reciprocal relationship with gene expression as described in the findings of Jiang et al. [32]. It is expected that individual genes may be differentially affected by CpG methylation and that only global analysis would be expected to reveal overall patterns likely to emerge.

### Canonical pathway, diseases and functions and network analyses by IPA

The 2147 genes with remarkable change in methylation (log2-fold change ≥ 2) were analyzed using the IPA software package. When using IPA, canonical pathways, which are based on the literature and are generated prior to data input, are the default settings. These pathways do not change upon data input and have a directionality-linked list of interconnected nodes. By contrast, networks are generated de novo based upon input data, lack directionality and contain molecules that are involved in a variety of canonical pathways.

The genes within the canonical pathways were ranked by the possibility parameter, i.e., the –log_10_(p) value in the corresponding pathway, and are presented in Table 5. The CREB1 gene, which is involved the neuropathic pain signaling pathway, was ranked first. The top networks ranked based on their ratios of methylated gene/total gene are listed in Table 6. Of the networks, HDAC2-related, tissue morphology, embryonic development, and organ development network was ranked first (Table 6). Among the networks, the cancer-related networks accounted for the majority (15/25) (Table 6), which indicates that the great difference between the TRAMP and control lies in organ development and cancer development.

**Table 5:** Top ten altered canonical pathways, sorted by − log_10_ (p) value via IPA

Pathways	− Log_10_ (p value)	Involved molecules
Neuropathic pain signaling in dorsal horn neurons	3.01	TACR1, GRM7, KCNN3, CAMK1D, MAPK1, GPR37, BDNF, GRM3, GRIA1, CREB1, TAC1, GRIN3A
Cardiomyocyte differentiation via BMP receptors	3.01	NKX2-5, MAP3K7, SMAD6, MEF2C, BMP10
cAMP-mediated signaling	2.75	ENPP6, ADCY2, RGS18, MAPK1, CAMK1D, PTGER3, GRM3, DUSP6, GNAI1, CHRM3, Cngb1, GRM7, FSHR, RGS10, CREB1, HTR1F, DRD3, PTGER4, PPP3CA
Estrogen biosynthesis	2.64	CYP4F8, CYP3A5, HSD17B7, CYP2C9, CYP2A6 (includes others), CYP51A1, CYP2C8
PXR/RXR activation	2.63	CYP3A5, ABCC2, INS, CYP2C9, CYP2A6 (includes others), INSR, PAPSS2, Ins1, CYP2C8
Wnt/β-catenin signaling	2.43	CDKN2A, GJA1, WNT3, APPL2, APC, SOX17, SOX2, FZD8, PPP2R1A, WNT7A, RARB, TLE4, MAP3K7, NR5A2, GSK3B
BMP signaling pathway	2.43	MAP2K4, NKX2-5, MAPK1, BMP8A, CREB1, MAP3K7, SMAD6, GREM1, BMP10
Factors promoting cardiogenesis in vertebrates	2.41	FZD8, SMAD2, NKX2-5, WNT3, BMP8A, MAP3K7, MEF2C, GSK3B, BMP10, APC
Glutamate receptor signaling	2.40	GRM7, SLC1A4, GRM3, GRIA1, SLC38A1, GRIP1, GRIK2, GRIN3A
Human embryonic stem cell pluripotency	2.39	SOX2, FZD8, SMAD2, WNT7A, WNT3, BDNF, BMP8A, SMAD6, GSK3B, NGF, APC, INHBA, BMP10
LPS/IL-1 mediated inhibition of RXR function	2.37	MAP2K4, GAL3ST2, ABCC2, CYP2C9, APOC2, NDST4, PAPSS2, IL1R2, TLR4, UST, CYP3A5, Sult1c2 (includes others), MAP3K7, NR5A2, CYP2A6 (includes others), GSTP1, MAOA, CYP2C8

**Table 6:** Top networks analyzed by IPA

Rank	Top diseases and functions	Score
1	Tissue morphology, embryonic development, organ development	38
2	Cell-to-cell signaling and interaction, cell signaling, cellular function and maintenance	38
3	Cell death and survival, cancer, cell morphology	37
4	Cancer, gastrointestinal disease, cell death and survival	35
5	Cancer, carbohydrate metabolism, small molecule biochemistry	33
6	Cancer, cell death and survival, cellular response to therapeutics	33
7	Lymphoid tissue structure and development, organ morphology, organismal development	30
8	Cancer, gastrointestinal disease, post-translational modification	29
9	Cancer, dermatological diseases and conditions, gastrointestinal disease	29
10	Cell morphology, digestive system development and function, nervous system development and function	28
11	Cancer, gastrointestinal disease, cell death and survival	26
12	Cancer, drug metabolism, energy production	26
13	Cell-to-cell signaling and interaction, nervous system development and function, cellular development	26
14	Cellular movement, cellular development, skeletal and muscular system development and function	24
15	Cell death and survival, cancer, cellular development	24
16	Hereditary disorder, inflammatory response, metabolic Disease	22
17	Cell morphology, nervous system development and function, tissue morphology	21
18	Cancer, organismal injury and abnormalities, reproductive system disease	21
19	Cellular compromise, cancer, cardiovascular disease	19
20	Cell-to-cell signaling and interaction, tissue development, hematological system development and function	17
21	Cancer, organismal survival, organismal injury and abnormalities	16
22	Cellular assembly and organization, cellular function and maintenance, embryonic development	16
23	Cancer, organismal injury and abnormalities, reproductive system disease	16
24	Cell cycle, cellular movement, cancer	16
25	Cancer, developmental disorder, hereditary disorder	16

**Fig. 6:**
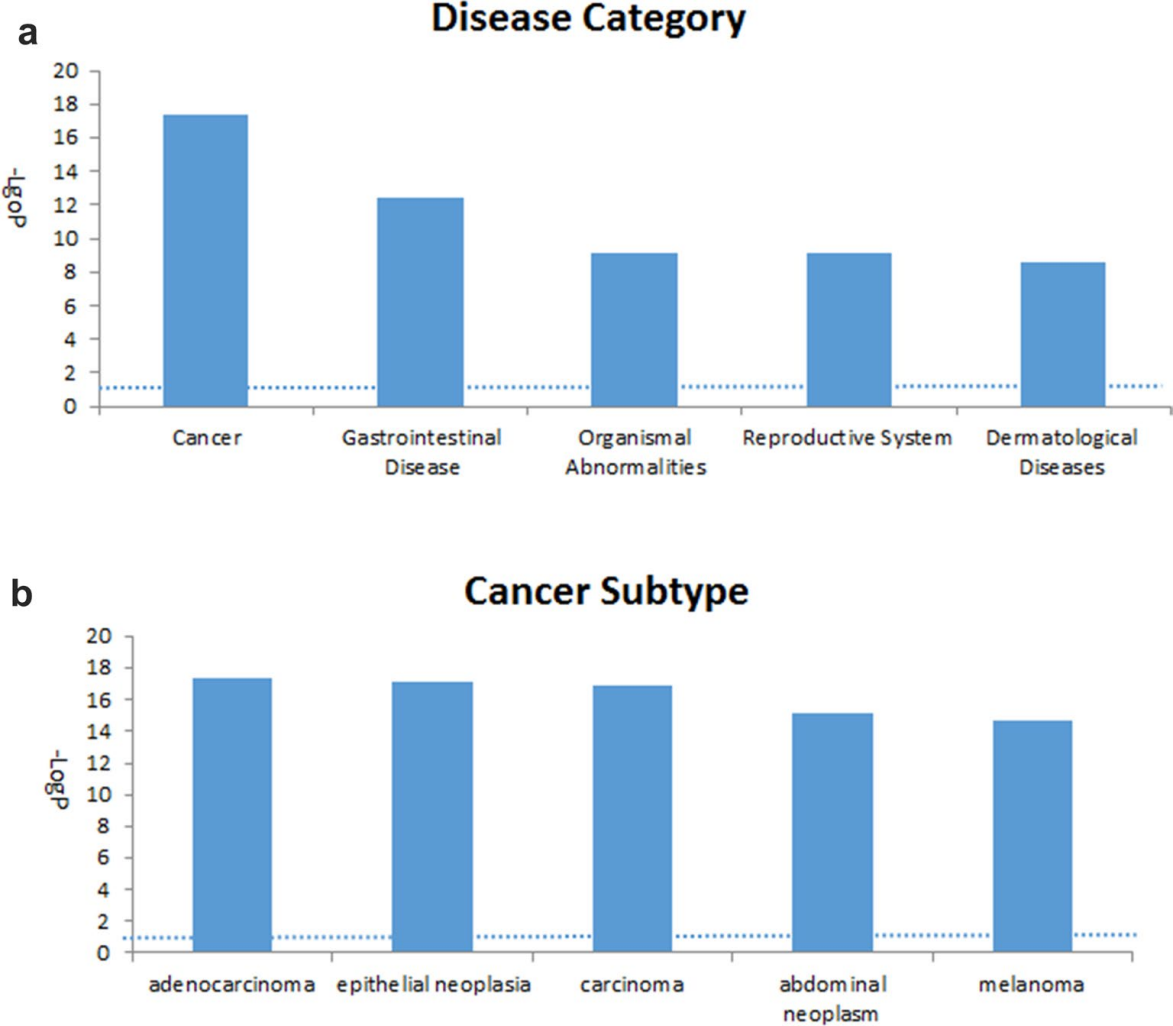
Top five associated disease categories (**a**) and top five cancer subtypes (**b**) analyzed by IPA

Diseases and functions refer to the most likely linked diseases or functions based on statistics. Similar to the network analysis, for the most associated disease based on the ranking of − log_10_p, cancer, gastrointestinal disease, organismal abnormalities, reproductive system disease and dermatological diseases were ranked within the top five (Fig. 6a). Of all cancer subtypes, adenocarcinoma ranked first (Fig. 6b), which was consistent with the TRAMP model, which is a model for prostate adenocarcinoma.

## Discussion

### Analysis of canonical pathway would provide further understanding of disease and information for the development of new therapeutic targets

**Fig. 7:**
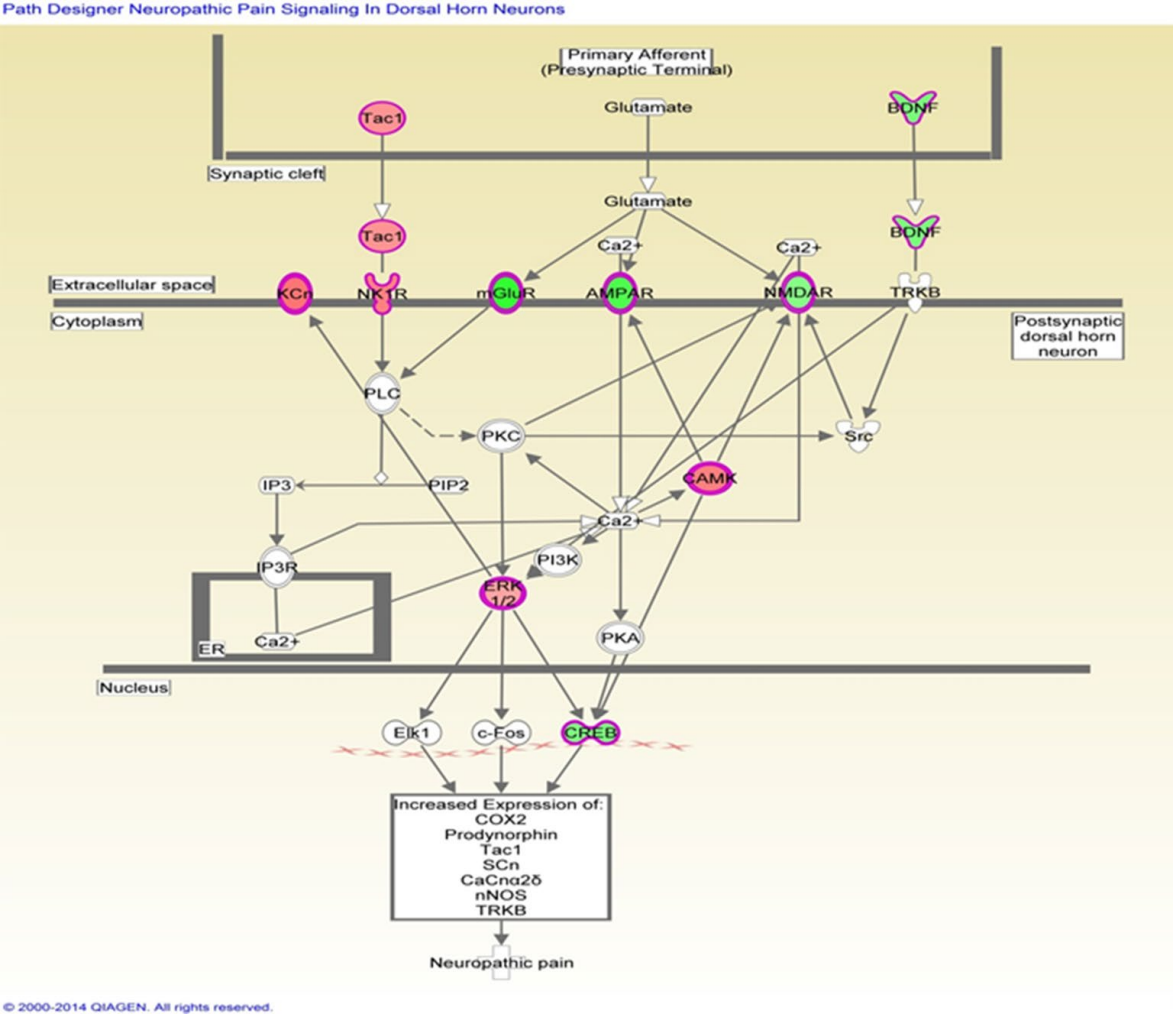
Genes mapped to the canonical neuropathic pain signaling pathway by IPA. Red, increased methylation; green, decreased methylation (for interpretation of the references to color in the figure legend, please refer to the online version of this article)

As shown in Fig. 7, the genes with significantly altered methylation in the top canonical pathway was the neuropathic pain signaling pathway, as mapped by IPA. This finding is consistent with Chiaverotti’s finding indicating that the most common malignancy in TRAMP is of neuroendocrine origin [33]. Table 7 lists the genes involved in this pathway that exhibited modified methylation. Among these, methylation of the CREB1 gene was found to be decreased by 2.274-fold (log2) by MeDIP-seq in TRAMP.

**Table 7:** Altered methylation genes mapped to the neuropathic pain signaling pathway by IPA

Symbol	Gene name	Log2-fold change	Type(s)
GRM3	Glutamate receptor, metabotropic 3	− 3.636	G-protein-coupled receptor
GRIA1	Glutamate receptor, ionotropic, AMPA 1	− 3.167	Ion channel
BDNF	Brain-derived neurotrophic factor	− 2.373	Growth factor
CREB1	cAMP-responsive element-binding protein 1	− 2.274	Transcription regulator
GRM7	Glutamate receptor, metabotropic 7	− 2.274	G-protein-coupled receptor
GRIN3A	Glutamate receptor, ionotropic, N-methyl-D-aspartate 3A	− 2.129	Ion channel
MAPK1	Mitogen-activated protein kinase 1	2.048	Kinase
TAC1	Tachykinin, precursor 1	2.408	Other
CAMK1D	Calcium/calmodulin-dependent protein kinase ID	2.855	Kinase
TACR1	Tachykinin receptor 1	2.855	G-protein-coupled receptor
KCNN3	Potassium intermediate/small conductance calcium-activated channel, subfamily N, member 3	3.119	Ion channel
GPR37	G protein-coupled receptor 37 (endothelin receptor type B-like)	3.855	G-protein-coupled receptor

CREB was first found to be closely related to cellular proliferation, differentiation and adaptive responses in the neuronal system [34, 35]. Subsequently, increasing evidence revealed that CREB is directly involved in the oncogenesis of a variety of cancers by regulating the immortalization and transformation of cancer cells [36, 37].

CREB is also found to modulate other carcinogenesis pathways. S100 calcium binding protein P (S100P) is a calcium-binding protein that is associated with cancer, and functional analysis of the S100P promoter identified SMAD, signal transducer and activator of transcription (STAT)/CREB and SP/KLF binding sites as key regulatory elements in the transcriptional activation of the S100P gene in cancer cells [38]. *Homo sapiens* lactate dehydrogenase c (hLdhc) was reported to be expressed in a wide spectrum of tumors, including prostate cancers, and this expression was shown to be regulated by transcription factor Sp1 and CREB as well as promoter CpG island (CGI) methylation [39, 40]. Decreased prostate tumorigenicity was found to be correlated with decreased expression of CREB and its targets, including Bcl-2 and cyclin A1.

Clinically, upregulation of CREB was found in various human cancer samples including prostate cancer, breast cancer, non-small-cell lung cancer and acute leukemia, whereas down-regulation of this gene manifested inhibition of some cancer cells [41].

All of these data indicate that CREB is highly associated with cancer therapy. Our study demonstrated that CREB gene methylation is significantly decreased in the TRAMP model, which suggests a new approach to prostate cancer prevention and therapy.

### Novel networks involving the methylation of target genes could provide new insights for prostate cancer

Compared with the canonical pathways, networks are generated de novo based upon input data and are able to more flexibly reveal the interactions of altered genes and functions. As it is impossible to analyze all networks listed in Table 6, four interesting networks were elaborated below (the higher the score is, the more genes with altered methylation are involved in the network). Among all these networks, many genes are known to be highly associated with tumor onset and progression, however, our insight into their methylation status alteration would reveal novel biomarkers for prostate tumorigenesis.

#### HDAC2-related network (score = 38)

**Fig. 8:**
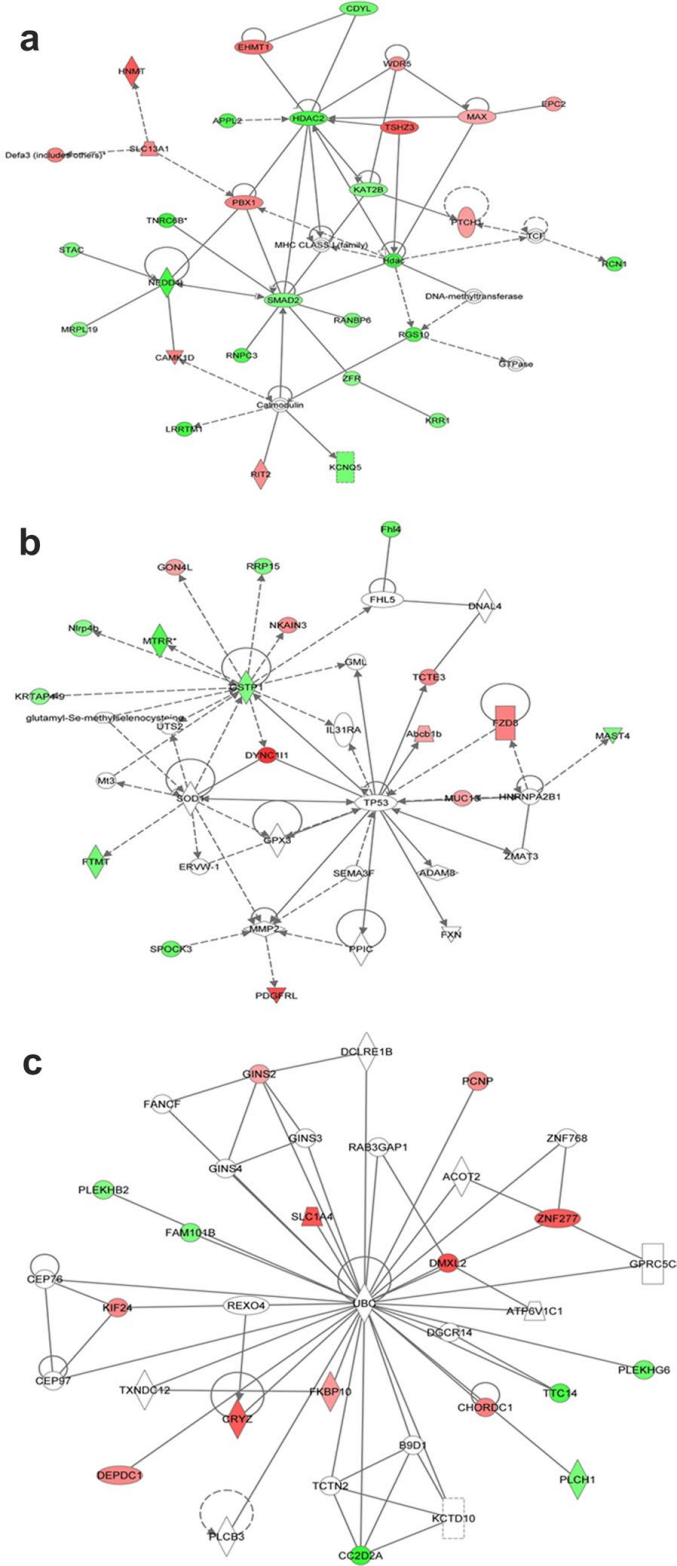
HDAC2 network (score = 38) (**a**), GSTP1 network (score = 16) (**b**), and UBC network (score = 16) (**c**), as determined by IPA. Red, increased methylation; green, decreased methylation (for interpretation of the references to color in the figure legend, please refer to the online version of this article)

The top network identified by IPA, was the HDAC2-related tissue morphology, embryonic development and organ development network (Table 6, Fig. 8a). In this network, the HDAC2 gene, a key member of HDAC, exhibited 3.274-fold (log2) decreased methylation in TRAMP. HDACs are responsible for the removal of acetyl groups from histones and play important roles in modulating the epigenetic process by influencing the expression of genes encoded by DNA bound to a histone molecule [42]. HDAC inhibitors have also been shown to reduce colonic inflammation [43], inhibit cell proliferation, and stimulate apoptosis, and these inhibitors represent a novel class of therapeutic agents with antitumor activity that are currently in clinical development [44, 45]. By upregulating histone H3 acetylation and p21 gene expression, long-term treatment with MS-275, an HDAC inhibitor, attenuated the progression of prostate cancer in vitro and in vivo [46]. Another HDAC inhibitor, OSU-HDAC42, also showed a chemoprevention effect on prostate tumor progression in the TRAMP model [47]. Our data suggest that the altered methylation of HDAC (3.274 log2-fold decrease) might be a novel, interesting target for prostate cancer treatment. Based on our MeDIP-seq results, HNMT in this network was increased by 3.703-fold (log2). In addition, based on our qPCR analysis, HNMT gene expression was reduced by 6.67-fold, which supports the likelihood of a role of HNMT in prostate cancer. However, although HNMT has been demonstrated to be associated with breast cancer [48] and liver cancer [49], little is known about its potential role in prostate cancer, making it another potential novel marker.

#### GSTP1-related network (score = 16)

GSTP1 expression is inactivated in prostate cancers [50–52], and this inactivation is associated with hypermethylation of GSTP1 CpG islands [51, 52]. Clinically, higher GSTP1 promoter methylation was found to be independently associated with the risk of prostate cancer [53]; therefore, the detection of hypermethylated GSTP1 in urine and semen samples can be a diagnostic marker of prostate cancer [54]. We also found that methylation of GSTP1 was an important factor involved in prostate cancer development. Interestingly, based on our data, the methylation of the GSTP1 gene was decreased 2.274-fold (log2) in TRAMP. Figure 8b demonstrates the decreased methylation of GSTP1. Based on comparisons of prostate samples from TRAMP and strain-matched WT mice, Mavis et al. [20] showed that promoter DNA hypermethylation does not appear to drive GST gene repression in TRAMP primary tumors. The above results support our finding that the methylation status of GSTP1 may differ in humans. DYNC1I1, which was also in the network, exhibited a 4.926-fold (log2) increase in methylation. In qPCR analysis, it indicates a 1.9-fold increase in gene expression. Although DYNC1I1 is significantly up-regulated in liver tumors [55] but not in prostate tumors, our findings suggest that it may be the next useful prostate cancer biomarker.

#### UBC-related network (score = 16)

Another interesting network was found surrounding the UBC gene (Fig. 8c); however, UBC itself was not identified by MeDIP-seq. The methylation of solute carrier family 1 member 4 (SLC1A4) and CRYZ was highly up-regulated (3.807 and 3.703 log2-fold increased, respectively). According to qPCR results, the expressions of SLC1A4 and CRYZ in TRAMP group were only 0.15- and 0.62-fold of WT group. SLC1A4 was found to be associated with human hepatocellular carcinoma [56], and CRYZ was proven to be involved in B-cell lymphoma 2 (BCL-2) overexpression in T-cell acute lymphocytic leukemia [57]. Although an association with prostate cancer was not found, our MeDIP–seq findings in the TRAMP model suggest that this association is possible.

#### Merged networks overlaid with IPA settings could even predict the direction of the relationship

**Fig. 9:**
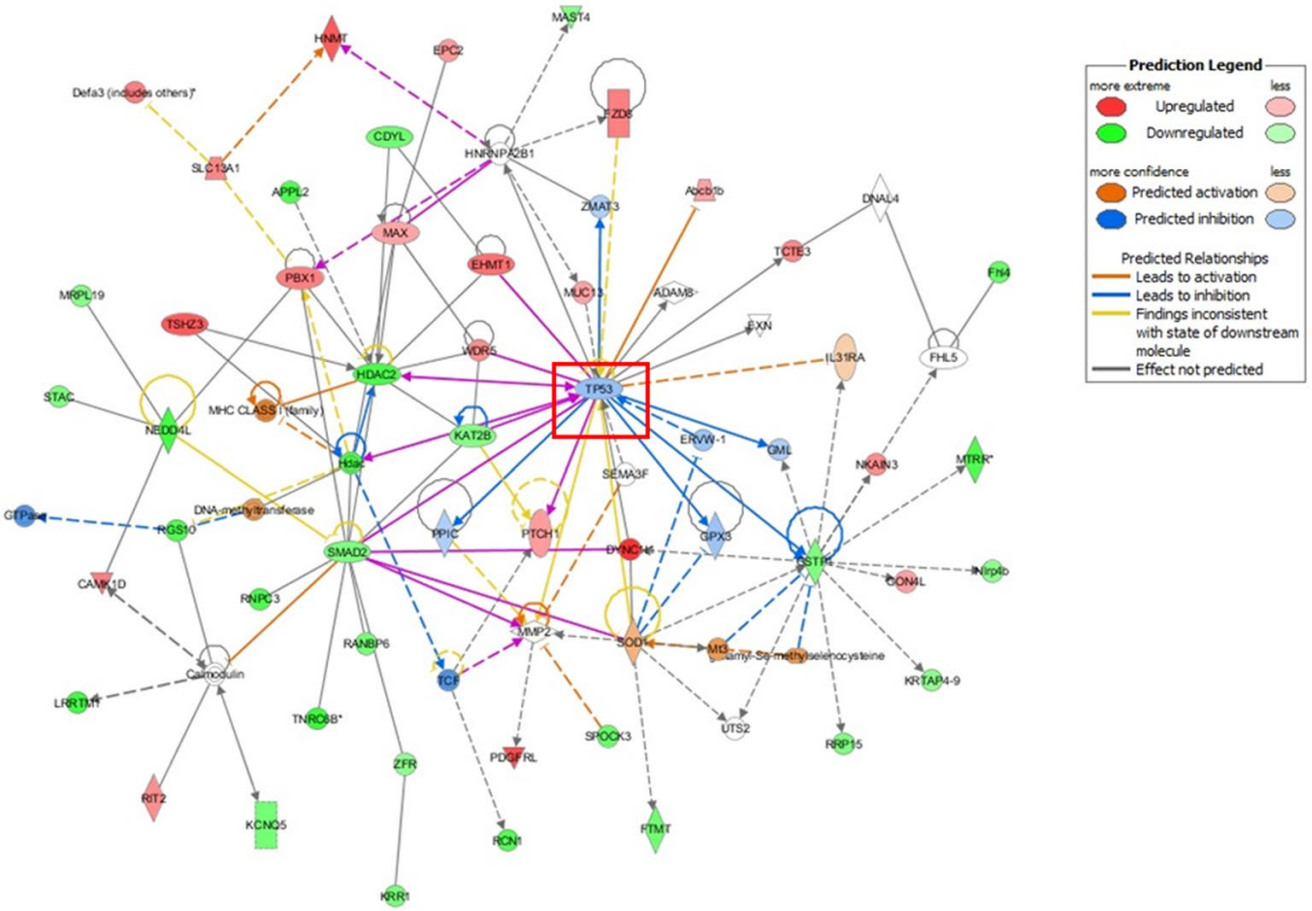
Merged network of the HDAC2 and GSTP1 networks, as determined by IPA. Red, increased methylation; green, decreased methylation (for interpretation of the references to color in the figure legend, please refer to the online version of this article)

When merging the two interesting networks HDAC2 and GSTP1 and overlaying the molecular activity predictor of IPA (Fig. 9), tumor protein 53 (TP53) was found to be located in the center of the novel network, indicating the potential important modulating function of TP53 on HDAC2 and GSTP1. TP53 is encoded by p53, a tumor suppressor gene located on chromosome 17p13, which is one of the most frequently mutated genes in multiple cancers [58–60]. TP53 acts as a transcription factor that mediates the response to various cellular stresses, most importantly, the DNA damage response [61]. TP53 has also been proven to play a crucial role in prostate cancer development and progression [62–64].

The interactions between GSTP1, HDAC and TP53 have been studied in prostate disease models. In prostatectomy specimens of 30 benign prostatic hyperplasia patients, the increase in TP53 expression at the same site was accompanied by an increase in GSTP1 expression [65]. In the 3 human prostate cancer cell lines DU-145, PC-3 and LNCaP, As_2_O_3_ was found to increase TP53 expression only in LNCap cells (without GSTP1 expression) but not in DU-145 and PC-3 cells (both cells expressed GSTP1) [66]. In LNCaP cells, the acetylation of human TP53 increased the binding of promoter fragments of the human P21 gene that contained a p53 response element and of the human HDAC2 protein [67].

Although the relationships between TP53 and HDAC2 as well as GSTP1 in prostate cancer have been elucidated, these relationships in the TRAMP model remain unknown. Our predicated interactions among these proteins in TRAMP suggest the possibility that TP53 influences the methylation of GSTP1 and HDAC2, which is a potential direction of future research.

## Conclusions

To the best of our knowledge, this is the first MeDIP-seq study to analyze the DNA methylation differences of prostate cancer by comparing TRAMP mice, an adenocarcinoma prostate cancer model, with wild-type C57BL/6 mice. Cancer, especially adenocarcinoma, is the most commonly associated disease. MSP and qPCR have been used to validate the findings of MeDIP-seq. Using this MeDIP-seq and IPA analysis, comparisons between the TRAMP and control samples reveal profound differences in gene methylation. The analysis of canonical pathways and networks has identified important biological functions and molecular pathways that may mediate the development of adenocarcinoma prostate cancer. CREB-, HDAC2-, GSTP1- and UBC-related pathways showed significantly altered methylation profiles based on the canonical pathway and network analyses. Studies on epigenetics, such as DNA methylation, suggest novel avenues and strategies for the further development of biomarkers targeted for treatment and prevention approaches for prostate cancer.

## Abbreviations

TRAMP: transgenic adenocarcinoma of the mouse prostate
MeDIP: methylated DNA immunoprecipitation
IPA: Ingenuity^®^ Pathway Analysis
CREB1: cyclic AMP (cAMP) response element-binding protein 1
HDAC2: histone deacetyltransferase 2
GSTP1: glutathione S-transferase 1
UBC: polyubiquitin-C
NRF2: nuclear factor (erythroid-derived 2)-like 2
MGMT: O6-alkylguanine DNA alkyltransferase
KLF6: Krueppel-like factor 6
DYNC1I1: dynein cytoplasmic 1 intermediate chain 1
SLC1A4: solute carrier family 1 member 4
Xrcc6bp1: XRCC6-binding protein 1
TTR: transthyretin
HNMT: histamine *N*-methyltransferase
CRYZ: crystallin zeta
